# The Effects of Rehabilitation Programs Incorporating Breathing Interventions on Chronic Neck Pain Among Patients with Forward Head Posture: A Systematic Review and Meta-Analysis

**DOI:** 10.3390/bioengineering12090947

**Published:** 2025-08-31

**Authors:** Seri Park, Kihyun Kim, Minbong Kang

**Affiliations:** 1 Seri Park Pilates Studio, 10, Teheran-ro 88-gil, Gangnam-gu, Seoul 06179, Republic of Korea; qmfcat@gmail.com; 2 Department of Physical Therapy, KyungWoon University, Gumi 39160, Republic of Korea; khpt05@ikw.ac.kr; 3 Department of Physical Therapy, Musculoskeletal Center, Daegu Medical Foundation K Hospital, 357, Chilgokjungang-daero, Buk-gu, Daegu 41452, Republic of Korea

**Keywords:** chronic neck pain, breathing exercise, forward head posture, systematic review, meta-analysis

## Abstract

The effectiveness of breathing interventions on postural alignment, pain reduction, and functional improvement in patients with forward head posture (FHP) and chronic neck pain remains uncertain. Previously conducted randomized controlled trials (RCTs) that involved breathing interventions were identified through searches of the PubMed, Cochrane Library, Web of Science, and Scopus databases. Studies were included if they applied diaphragmatic breathing, breathing muscle training, or feedback breathing exercises for at least 2 weeks to chronic neck pain (duration ≥ 3 months) and/or forward head posture. The craniovertebral angle (CVA), the visual analog scale (VAS), and the neck disability index (NDI) were the primary outcome measures. The results showed that breathing interventions had a moderate effect size in terms of improving the CVA. Limited effects were observed for pain reduction, and improvements in neck disability approached statistical significance. However, despite these positive findings, the overall evidence was rated as ‘very low certainty’ in the GRADE assessment, primarily due to high heterogeneity among studies, limited sample sizes, and the potential for unit-of-analysis errors in diagnosis-based subgroup analyses. Consequently, their overall effectiveness in chronic neck pain was limited. Future research is needed to explore a multidisciplinary approach to neck pain using standardized protocols and larger samples.

## 1. Introduction

Chronic neck pain (CNP) is a commonly observed musculoskeletal disease that causes functional decline and reduced quality of life for many patients. The increase in sedentary lifestyles in modern society and the prominence of computer work and smartphone use have made it very common for forward head posture (FHP) to occur in conjunction with CNP [1,2], which suggests that these two conditions may share complex pathophysiological mechanisms that influence each other [3,4]. CNP and FHP can be caused and aggravated by an increased mechanical load on the cervical region as well as by factors such as abnormal muscle activation patterns and nervous system sensitization.

Recent studies have focused on the impact of poor posture, particularly FHP, on breathing function. Forward head posture restricts the movement of the thorax and reduces the efficiency of the diaphragm, the primary respiratory muscle [5,6,7,8,9]. It can also lead to an overuse of accessory respiratory muscles and thereby increase tension in the cervical and scapular muscles. There exists a direct physiological mechanism between these changes in breathing patterns and neck pain. Inefficient breathing can reduce oxygen supply to the muscles and lead to the accumulation of metabolic byproducts, which can cause or exacerbate pain, and chronic excessive activity of the accessory respiratory muscles can lead to fatigue and pain in the cervical muscles [10,11]. Therefore, therapeutic breathing interventions have been theoretically proposed not only for improving breathing function but also for alleviating the fundamental pathophysiological link between CNP and FHP [12,13].

Conventional rehabilitation programs for CNP and FHP have primarily focused on strengthening, stretching, and postural correction exercises. While these approaches have exhibited some effectiveness, their therapeutic outcomes may be limited by the association with breathing function being overlooked. To date, there is a lack of meta-analyses that systematically synthesize and quantify the comparative effectiveness of including breathing interventions versus excluding them in rehabilitation programs for CNP and FHP.

The present study was conducted to fill this important gap in the literature and to quantitatively evaluate the incremental value gained by integrating breathing interventions into rehabilitation programs. Previous meta-analyses have focused exclusively on either CNP or FHP or have only analyzed the effects of specific types of exercise, without clearly comparing the effects of incorporating breathing interventions into rehabilitation programs [14,15,16]. We aimed to address these limitations by systematically analyzing how the inclusion of breathing exercise interventions alters the effectiveness of rehabilitation programs. Our objective was to propose the most effective treatment strategies for patients with CNP and FHP and to provide clinicians with the information necessary for evidence-based decision-making. Therefore, this study holds significant clinical relevance in both the theoretical and practical aspects of the field of CNP and FHP rehabilitation.

The purpose of this study was to systematically evaluate and obtain quantitative evidence on pain and postural improvement among patients with CNP and FHP when breathing exercise interventions were included in rehabilitation programs compared to when they were not. Through this, we aimed to offer scientific guidelines for the optimal composition of rehabilitation programs for patients with CNP and FHP. Previous meta-analyses have focused exclusively on either CNP or FHP or have only analyzed the effects of specific types of exercise, without comparing the effects arising from the inclusion of breathing interventions [14,15,16]. The present study was conducted to fill this important gap in the literature. The aim of this study was to address these limitations by systematically analyzing how the inclusion of breathing exercise interventions alters the effectiveness of rehabilitation programs and thereby identify the most effective treatment strategies for patients with CNP and FHP and provide clinicians with the information necessary for evidence-based decision-making. Therefore, this study holds significant clinical relevance in both the theoretical and practical aspects of CNP and FHP rehabilitation.

## 2. Materials and Methods

This systematic review has been registered on PROSPERO with the protocol number CRD420251032380, and the report follows the Preferred Reporting Items for Systematic Review and Meta-Analyses (PRISMA) [17]. The review was conducted in line with the updated guidelines for systematic reviews from the Cochrane Back and Neck Group [18].

### 2.1. Deviations from the Protocol

The initial protocol for this systematic review was slightly modified due to limitations identified during the literature search and eligibility screening process. We initially aimed to analyze the clinical effectiveness of breathing exercises as a standalone intervention for patients with neck pain. However, due to insufficient studies addressing breathing exercises alone, the research question was redefined to evaluate the effectiveness of rehabilitation programs that incorporate breathing interventions. This refining aimed to provide a more meaningful comparison and comprehensive presentation of the current evidence. After refining the research question, the clinical outcome variables assessing pain (visual analog scale [VAS] or numeric rating scale [NRS]), functional disability (Nerve Disability Index [NDI]), and postural alignment (craniovertebral angle [CVA]), as well as all relevant inclusion criteria, were updated to reflect this new comparison-based approach. These inevitable adjustments were made to enhance the scientific validity of this study and to reflect the characteristics of the available literature for optimal results. This methodological adjustment, while necessary given the available literature, introduced complexity in isolating the specific effects of breathing interventions from other concurrent treatments. We acknowledge that this scope expansion may have reduced the specificity of our findings regarding the independent therapeutic value of breathing interventions. The observed high heterogeneity in pain and functional outcomes may be partially attributed to this methodological complexity.

### 2.2. Search Strategy

A total of five randomized controlled trials (RCTs) (204 participants) were included in this meta-analysis [19,20,21,22,23]. A comprehensive search strategy was developed to identify all relevant studies that have evaluated the effectiveness of breathing interventions in treating chronic neck pain and forward head posture. Our systematic search included the PubMed, Cochrane Library, Web of Science, Embase, CINAHL, and Scopus databases. We used combinations of subject headings and free-text terms and adapted them to each database’s specific indexing system.

The search terms were organized into three main concepts using Boolean operators as follows. (Population): (“Neck pain” OR “Cervical pain” OR “Chronic neck pain” OR “Forward head posture” OR “FHP” OR “Upper crossed syndrome” OR “Cervical spine” OR “Cervical dysfunction” OR “Text neck” OR “Tech neck”) AND (Intervention): (“Breathing exercise” OR “Respiratory exercise” OR “Diaphragmatic breathing” OR “Deep breathing” OR “Breathing training” OR “Breathing therapy” OR “Respiratory rehabilitation” OR “Breathing pattern” OR “Respiratory muscle training” OR “Inspiratory muscle training” OR “Feedback breathing” OR “Balloon breathing”) AND (Study Design): (“Randomized controlled trial” OR “RCT” OR “Controlled trial” OR “Clinical trial” OR “Randomized” OR “Random allocation”).

The search was conducted on 4 June 2025, with no restrictions on the publication date to ensure comprehensive coverage of all available evidence. English language publications were included to maximize the comprehensiveness of the search and ensure accessibility of the research findings. Appendix A shows the search strategy.

### 2.3. Eligibility Criteria

**Figure 1:**
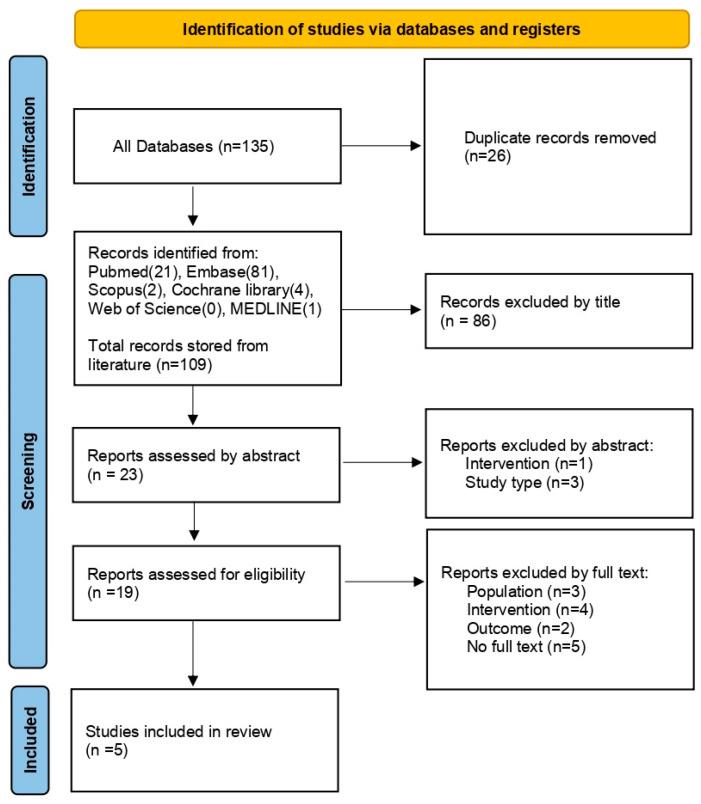
Flowchart illustrating the study selection process based on the latest PRISMA guidelines.

The eligibility criteria were based on the PICOS (population, intervention, comparator, outcome, and study design) framework [24]. PICOS eligibility inclusion/exclusion criteria are provided in Table 1. The study selection process is depicted in Figure 1, following PRISMA guidelines.

### 2.4. Rationale for the Criteria

The inclusion criteria were designed to ensure homogeneity in the target population while maintaining clinical relevance. The minimum intervention duration of two weeks was established based on evidence that neural adaptation and measurable changes in breathing patterns require at least this timeframe. The focus on nonspecific chronic neck pain was based on the most common presentation in clinical practice and to avoid confounding factors associated with specific pathological conditions. Active control groups were prioritized to reflect real-world clinical scenarios in which patients typically receive some form of treatment, rather than no intervention. We expected this approach to enhance the external validity and clinical applicability of the findings and also allow for assessing the specific contributions of breathing exercises to treatment outcomes.

### 2.5. Data Extraction

Two reviewers (SR and MB) independently extracted data from the articles included in the review, then compared the data extracted and created a single file. Details of the included studies are presented in Appendix B.

### 2.6. Methodological Quality Assessment

To assess the strength and quality of the evidence, the two reviewers (SR and MB) assessed the risk of bias of each study using the Cochrane Collaboration’s risk of bias assessment tool [25]. This tool assesses bias across five domains: bias arising from the randomization process, bias due to deviations from intended interventions, bias due to missing outcome data, bias in outcome measurements, and bias in selecting the reported results. The five domains of risk were rated as “high”, “low”, or “unclear”, and the overall risk of bias was determined.

### 2.7. Data Analysis

A meta-analysis and statistical analyses were conducted using the meta package in the R software program, version 4.3.2 [26]. To estimate the effect sizes of the pain and functional disability outcomes, the standardized mean differences (SMDs) and 95% confidence intervals (CIs) were calculated for the continuous outcomes of each study, and a random-effects model was applied. In line with Cohen’s guidelines, SMD values of 0.2–0.5 were interpreted as small effect sizes, 0.5–0.8 as medium effect sizes, and greater than 0.8 as large effect sizes [27]. In all the analyses, *p* < 0.05 was considered statistically significant. After the primary analysis, subgroup analyses were performed to explore whether the effects of breathing interventions differed depending on patient characteristics. The patients were divided into FHP and CNP groups based on diagnosis type, into groups under 30 years of age and 30 years or older based on age, and into groups with an initial NDI score of less than 20 and a score of 20 or higher based on the level of neck functional disability. Through the subgroup analyses, we explored the differential effects of breathing interventions on pain (VAS), postural alignment (CVA), and functional disability (NDI). Heterogeneity among the studies was assessed using Higgins’ I^2^ statistic and *p*-values [28]. When the I^2^ value exceeded 50%, substantial heterogeneity was considered present, and a random-effects model was applied. Publication bias was assessed using both visual and statistical methods. A funnel plot of the outcome values was initially created to visually examine asymmetry, and Duval and Tweedie’s trim-and-fill method was then applied to estimate the potential adjustment effect for publication bias. The number of studies included in the meta-analysis for each outcome was small (VAS: 3 studies, NDI: 4 studies, CVA: 4 studies) because of which the statistical power of Egger’s regression asymmetry test was deemed insufficient, and the test was not performed. Finally, a series of leave-one-out analyses were conducted to determine sensitivity. This involved repeating the meta-analysis and excluding one study each time to evaluate the influence of the individual studies on the overall effect estimates and to confirm the robustness of the results.

### 2.8. Subgroup Analysis Justification

In this study, three predefined subgroup analyses were conducted to explore the potential sources of heterogeneity: (a) diagnosis category (CNP vs. FHP), (b) baseline NDI score, and (c) age. The methodological rationale for each criterion was as follows.

(a) Diagnosis category: CNP vs. FHP

In this study, CNP and FHP were predefined subgroups, and for individual RCTs that recruited both diagnostic groups of patients together, the CNP and FHP results reported were each included as separate effect sizes. In other words, when VAS, CVA, and NDI were separately reported by a diagnostic group within the same study, a single study was entered as two meta-analysis comparison units.

This approach is consistent with the situation delineated in the Cochrane Handbook v6.5 Section 23.3.4, which states, “double-counts the participants, leading to a unit-of-analysis error” [29]. It is acknowledged that this represents a significant methodological limitation that could lead to overestimation of precision and introduces potential bias into the results. Although a random-effects model was employed in all analyses to address residual heterogeneity and correlation, this approach does not fully resolve the unit-of-analysis error. Consequently, results from diagnosis-based subgroup analyses should be interpreted with considerable caution, and future studies should employ robust variance estimation or multilevel meta-analysis techniques to properly handle dependent effect sizes.

The subgroup analyses by age (<30 years vs. ≥30 years) and functional disability level (NDI < 20 points vs. ≥20 points) were designed to include only a single effect size per study, which allowed for avoiding the double-counting issue that occurred when analyzing studies by diagnosis. Age and NDI were managed as study-level variables; this ensured that each study contributed only one unit of analysis, and overlapping multiple comparison results were not derived from the same study. By applying a random-effects model, residual correlations and heterogeneity were simultaneously controlled. Through these procedures, the unit-of-analysis error was limited to the diagnosis-based subgroups, and complete independence of the analysis units was maintained in the age and functional disability subgroups.

(b) Level of neck disability: NDI ≥ 20 points vs. <20 points

In this meta-analysis, a cutoff score of 20 points on the NDI was used to classify subgroups according to the participants’ baseline level of neck disability, with NDI < 20 points indicating mild disability and NDI ≥ 20 points indicating moderate or greater disability. This criterion was established based on clinical interpretation guidelines for the NDI and on prognostic studies [30].

Vernon and Mior, the original developers of the NDI, classified 0–14 points as indicative of mild disability, 15–34 points as indicative of moderate to severe disability, and 35 points or higher as indicative of severe disability. According to this classification, 20 points fall within the moderate disability range. In addition, 20 points correspond to 40% of the total score of 50 and suggest that neck pain-related functional disability has reached a clinically meaningful level [31]. Patients with an NDI score of 20 points or higher present a level of disability that requires therapeutic intervention, and forming subgroups based on this threshold ensures both clinical clarity and statistical validity [32]. In fact, multiple studies have reported that patients with NDI ≥ 20 points exhibit more pronounced pain, postural abnormalities, and functional impairments than those with lower NDI scores, and they also demonstrate different responsiveness to treatment [33].

Prognostic studies have repeatedly confirmed the meaningful cutoff value of NDI ≥ 20 points. Atherton et al. reported that an NDI > 19 points was a strong independent predictor of persistent neck pain one year post diagnosis and that those patients were more than three times at risk of nonrecovery [34]. Similarly, a study by Miettinen and Leino showed that whiplash injury patients with an initial NDI score of 20 or higher had more than a sevenfold increased likelihood of failing to recover after three years, and NDI was the only significant predictor in a multivariate analysis [35].

Considering the aforementioned NDI-related studies, the NDI score of 20 serves as a cutoff point for determining both treatment responsiveness and prognosis in terms of pain and functional disability. Furthermore, because it is consistent with the cutoff point commonly used in existing clinical studies and guidelines, classification based on the NDI score increases the clinical applicability and generalizability of the results [30].

(c) Age classification: <30 years vs. ≥30 years

Age is a physiological moderating variable that can influence patients’ responsiveness to breathing interventions. In this study, subgroups were classified using 30 years as the cutoff. This criterion was based on the following rationale. Age affects respiratory muscle function and the adaptive capacity of the musculoskeletal system. In adults, lung capacity and muscle strength peak between the ages of 20 and 30, after which they begin to gradually decline. After the age of 30, breathing efficiency decreases because of reduced rib cage flexibility and a decline in diaphragmatic muscle fibers [36,37,38,39,40]. In this study, the age subgroups of <30 years and ≥30 years were established to explore differences in the effects of breathing interventions with regard to physiological aging. This classification was thus based on scientific evidence regarding age-related changes in breathing function.

Our multilayered subgroup analyses aligned with the procedures recommended in the Cochrane Handbook for exploring heterogeneity and enhancing the clinical validity of result interpretations. The data for each subgroup were treated as independent units of analysis, and no double counting of the same participants occurred. This ensured the statistical independence of the analysis and the reliability of the results.

### 2.9. Certainty of Evidence

The Grading of Recommendations Assessment, Development and Evaluation (GRADE) method was used to appraise the overall certainty of the pooled evidence and the evidence for each outcome (VAS, NDI, and CVA) [41]. The quality of evidence was assessed as “high”, “moderate”, “low”, or “very low” by two independent reviewers (SR and MB). Every article included in the systematic review was rated based on the criteria described by Guyatt et al. [42], namely risk of bias, inconsistency, indirectness, imprecision, and publication bias. Moreover, as suggested by Guyatt et al. [43], the quality of evidence was downrated when the total sample size was low (<24 participants), as this could lead to imprecision.

## 3. Results

### 3.1. Study Selection

The initial database search yielded 135 records (PubMed, n = 21; Embase, n = 87; Scopus, n = 2; Web of Science, n = 5; Cochrane, n = 15). Following deduplication procedures, 109 records were retained for preliminary evaluation based on their titles and abstracts. Subsequently, a detailed assessment resulted in 19 studies that warranted a comprehensive full-text examination. The complete workflow of the selection methodology is presented in Figure 1.

All records retrieved from the database search were imported into Endnote (Clarivate Analytics, USA), a publication management software. The titles and abstracts were screened independently by two reviewers (SR and MB) according to the eligibility criteria. Disagreements were resolved by discussion between the two reviewers. A third reviewer (KH) was available in the case of further disagreement. In the second stage of screening, the two reviewers examined the full texts to determine the final eligibility of the studies.

### 3.2. Characteristics of the Included Studies

The five studies ultimately included were all RCTs that involved adults with forward head posture, chronic neck pain, or upper crossed syndrome [19,20,21,22,23]. The total number of participants in these studies ranged from 24 to 60, and the mean ages ranged from 23.9 years (±2.3) to 56.5 years (±11.4)—that is, young to middle-aged adults. Most studies included a mixed-gender population, but some focused on a specific gender. For example, Jeong and Lee [23] targeted 37 young men in their 20s and 30s, and Mosallaiezadeh et al. [22] focused on 30 women with chronic neck pain. In a study conducted by Metikaridis et al. [20], approximately 95% of the participants were women.

The primary diagnoses of the participants were nonspecific chronic neck pain, forward head posture, or upper crossed syndrome, and they met specific criteria (e.g., CVA less than 52°, FHP greater than 5 cm, NDI 28–45%, VAS ≥ 3). The exclusion criteria included severe underlying pathologies (tumor, infection, severe injury, herniated disk, etc.), severe mental illnesses, and pregnancy.

The breathing interventions used in the included studies varied. The primary method was diaphragmatic breathing, and basic diaphragmatic breathing was implemented in two studies [20,23]. Resistance breathing was performed using equipment in Mosallaiezadeh et al.’s study [22]; a 2.5 kg weight was placed on the abdomen of each patient in a supine position with 40 degrees of trunk flexion. In Kang et al.’s study [19], feedback breathing training was conducted using the SPIROTIGER^®^ device, and in Dareh-Deh et al.’s study [21], balloon breathing exercises were performed, which involved exhaling into a balloon. The intervention duration ranged from two to eight weeks, and the frequency varied from one to five times per week.

All studies involved active control groups that underwent interventions other than breathing exercises. The control group interventions included McKenzie exercises, shoulder stabilization exercises, standard physiotherapy, a combination of resistance exercises and stretching, and stress management programs. CVA, VAS, and NDI scores were the main outcome variables used in all the studies. CVA was used as an objective measurement index of FHP, and in all studies, it was quantified on angle measurement software using photographs. The NDI was used as a standardized tool to evaluate the level of functional disability, and the baseline NDI scores of the study participants ranged from 8.66 points to 29.93 points, indicating mild to moderate disability. The VAS was consistently used in all studies as a tool to evaluate subjective pain levels.

The postintervention measurements of the intervention groups showed statistically significant improvements compared to the baseline values in all studies, and they also showed significant improvements compared to the control group in most studies. Consistent CVA improvement was observed in all studies, suggesting the direct effect of breathing intervention on postural alignment.

Two studies included additional outcome variables to evaluate the multidimensional effects of breathing intervention. Dareh-Deh et al. [21] measured breathing-related indicators such as diaphragmatic muscle activation, breathing balance, and respiratory rate to confirm the direct physiological effects of the intervention. Metikaridis et al. [20] included stress-related physiological indicators in their study to evaluate the effect of the breathing intervention on the autonomic nervous system.

In Dareh-Deh et al.’s study [21], the breathing intervention group showed significant differences in diaphragmatic muscle activation (*p* = 0.03), breathing balance (*p* = 0.01), and respiratory rate (*p* = 0.02) when compared to the control group. This result directly proved that the balloon breathing intervention was effective at improving breathing patterns. However, these secondary outcome variables were not superior to general treatment programs, suggesting that the effects of the breathing intervention were concentrated in specific areas.

The included studies consistently showed that breathing interventions can have positive effects on postural alignment, pain, and functional disability, which are the main symptoms of FHP and CNP. Consistent improvement trends were seen by the various age groups and genders with different intervention types, suggesting the wide applicability of breathing interventions. In particular, the effects were confirmed in various age groups comprising young adults to middle-aged people, providing basic data on the differences in treatment responses according to age.

### 3.3. Risk of Bias

**Figure 2:**
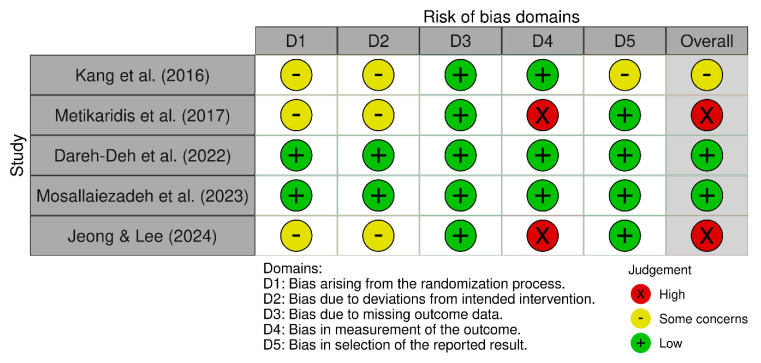
Risk of bias summary plot of included studies [19,20,21,22,23].

**Figure 3:**
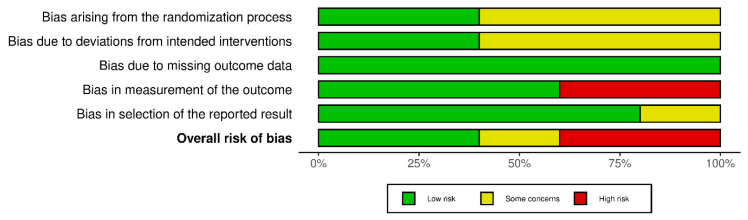
Summary of the literature quality assessment.

The risk of bias was assessed for the five included RCTs using the RoB 2.0 tool. Among them, two studies were identified as having a high risk of bias [20,22], one study was identified as having some concerns [39], and the remaining two studies were identified as having a low risk of bias [21,22] (Figure 2). Figure 3 summarizes the results of the risk of bias assessment for each study by domain. Overall, in some studies, risk factors were found in areas such as randomization procedures or a lack of blinding in outcome assessments, but most studies showed relatively good methodological quality.

#### Domain-Specific Bias Analysis and Impact on Outcome Interpretation

Bias arising from the randomization process (D1) showed mixed results across studies. While two studies demonstrated low risk [21,22], three studies raised some concerns [19,20,23]. The studies that have given rise to concerns pertain to domains in which it is suspected that the enrolled investigators or participants were cognizant of the allocation. This prompts concerns regarding the potential for subjective intervention by participants or investigators, which could substantially influence treatment effect estimates for subjective outcomes such as pain and disability.

Concerns have been raised in three studies [19,20,23] regarding bias due to deviations from intended interventions (D2). However, there is a paucity of information regarding the blinding process during the intervention period, as both studies fail to explicitly address whether the interventionist or participants were blinded to their assigned intervention. This limitation is hypothesized to have exerted divergent effects on the outcome measures. While objective postural assessment (CVA) demonstrates a diminished susceptibility to performance bias in comparison with self-reported pain (VAS) and disability (NDI), it remains vulnerable to rater bias. Furthermore, the implementation of questionnaire-based measures, including the VAS for pain and the NDI for disability, may encompass subjective factors. The absence of participant blinding could have led to the overestimation of treatment effects on subjective outcomes through expectancy bias and the placebo effect.

Bias due to missing outcome data (D3) was well-managed across all included studies, with all five studies rated as low risk. This demonstrates appropriate handling of participant dropout and missing data through intention-to-treat or similar approaches.

In the domain of bias in measurement of the outcome (D4), two studies [20,23] were identified as high risk. It has been posited that studies that were assessed as high risk may have lacked the implementation of blinding measures for assessors responsible for subjective outcomes. This potential deficiency may have resulted in an overestimation of treatment effects.

Bias in the selection of the reported result (D5) was generally well-controlled, with four studies showing low risk [20,21,22,23] and one showing some concerns [19].

Overall assessment results identified two studies as high risk [20,23], one study as of some concern [19], and two studies as low risk [21,22]. The high-risk studies were downgraded due to concerns about measurement bias, which may influence the interpretation of subjective results.

### 3.4. Meta-Analysis Results

#### Effects of Breathing Exercises on VAS, CVA, and NDI Among CNP and FHP Patients

**Figure 4:**
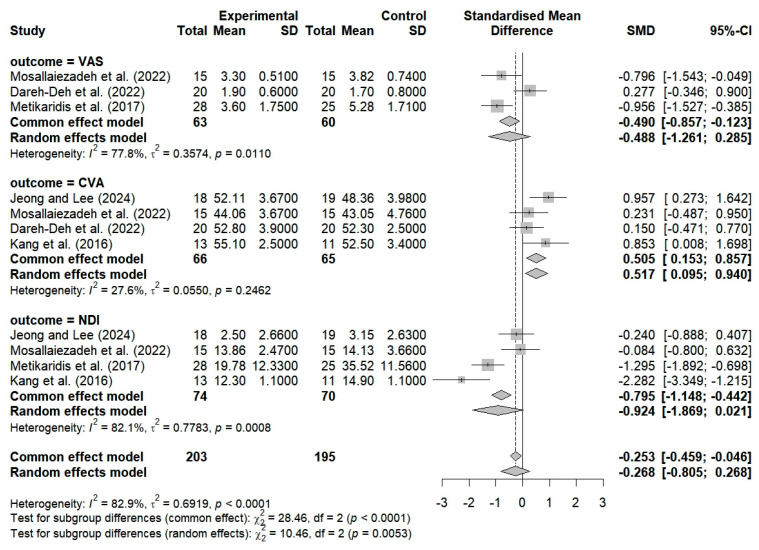
Effect of breathing exercise on VAS, CVA, NDI among CNP and FHP patients [19,20,21,22,23].

The VAS was used in three studies to assess the effects of the breathing interventions on changes in the pain levels of patients with FHP and CNP. The findings showed that the breathing interventions had no significant effects on pain, and high heterogeneity was observed among the studies (SMD = −0.488; 95% CI: −1.261 to −0.285; I^2^ = 77.8%, *p* = 0.0110, n = 123) (Figure 4).

CVA was used in four studies to evaluate the effects of breathing interventions on changes in neck alignment among patients with FHP and CNP. The findings showed that the breathing interventions improved neck alignment, with moderate heterogeneity among the studies (SMD = 0.505; 95% CI: 0.153 to 0.857; I^2^ = 27.6%, *p* = 0.2462, n = 131) (Figure 4).

Four studies evaluated the effects of breathing interventions on the neck NDI scores of patients with FHP and CNP. The breathing interventions did not significantly change the patients’ NDI scores, and high heterogeneity was observed among the studies (SMD = −0.924; 95% CI: −1.869 to 0.021; I^2^ = 82.1%, *p* = 0.0008, n = 144) (Figure 4).

The overall SMD was −0.268 (95% CI: −0.805 to 0.068; I^2^ = 82.9%, *p* < 0.0001, n = 398), which indicated that the breathing exercises had a marginal tendency to enhance the patients’ outcomes compared to the control group. However, this difference was not statistically significant (*p* > 0.05).

### 3.5. Subgroup Analyses

#### 3.5.1. The Subject-Specific Effects of the Interventions on VAS, CVA, and NDI Among Patients with FHP and Neck Pain

**Figure 5:**
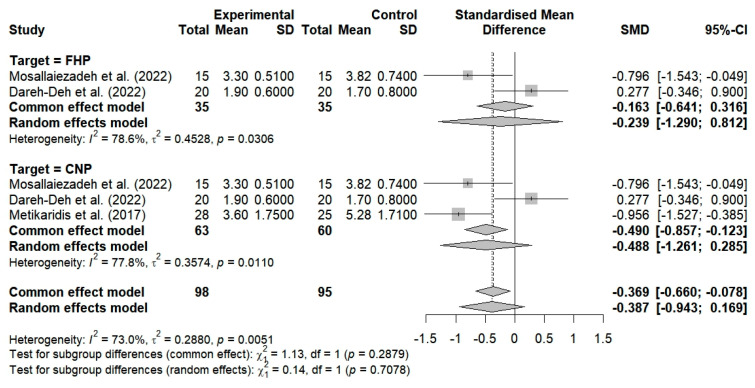
Effect of breathing exercise on VAS in each patient with CNP and FHP [20,21,22].

Two studies included breathing exercises for FHP, but they failed to demonstrate a substantial improvement in pain levels (SMD = −0.239; 95% CI: −1.290 to 0.812; I^2^ = 78.6%, *p* = 0.0306, n = 70) (Figure 5). Three studies included breathing exercises for patients with neck pain; no substantial improvement in pain was observed (SMD = −0.488; 95% CI: −1.261 to 0.285; I^2^ = 77.8%, *p* = 0.0110, n = 123) (Figure 5). The random-effects model resulted in an overall SMD of −0.387 (95% CI: −0.943 to 0.169; I^2^ = 73%, *p* = 0.0051, n = 193). This finding indicates that the breathing exercises resulted in a modest improvement among the FHP and CNP patients in comparison to the control group. However, this improvement was not statistically significant at the *p* > 0.05 level.

**Figure 6:**
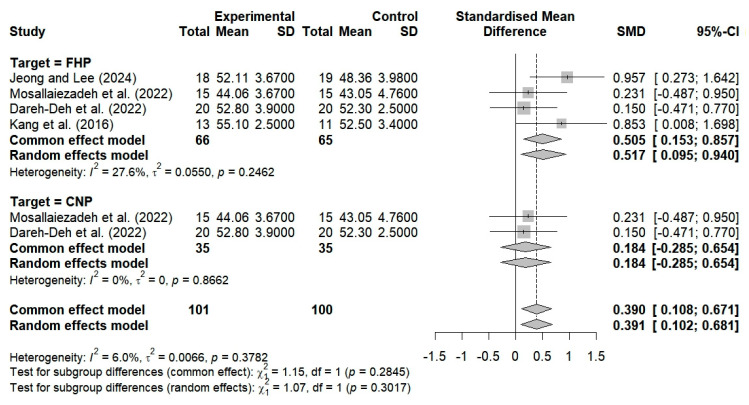
Effect of breathing exercise on CVA in each patient with CNP and FHP [19,21,22,23].

A total of four studies evaluated the impact of breathing exercises on alterations in neck alignment among individuals with CVA in FHP. These studies revealed substantial changes in alignment (SMD = 0.517; 95% CI: 0.095 to 0.940; I^2^ = 27.6%, *p* = 0.2462, n = 131) (Figure 6). Two studies investigated the impact of breathing exercises on patients with neck pain, yielding nonsignificant outcomes (SMD = 0.184; 95% CI: −0.285 to 0.654; I^2^ = 0%, *p* = 0.8662, n = 70) (Figure 6).

The implementation of breathing exercises among patients with both FHP and CNP resulted in substantial alterations in CVA, with an overall SMD of 0.391 (95% CI: 0.102 to 0.681; I^2^ = 6%, *p* < 0.3782, n = 201), when compared to the control group (*p* < 0.05).

**Figure 7:**
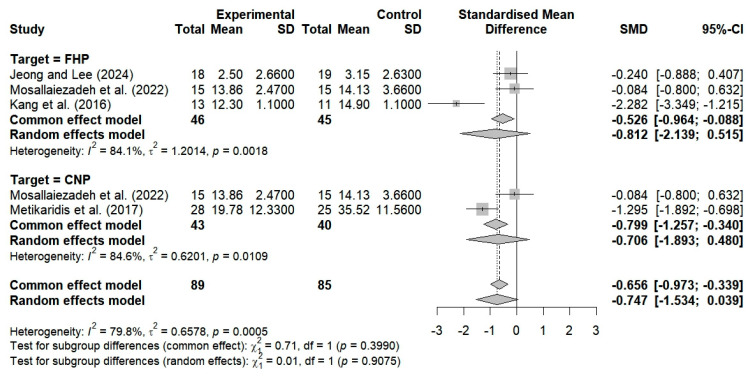
Effect of breathing exercise on NDI in each patient with CNP and FHP [19,20,22,23].

A total of three studies examined the impact of breathing exercises on NDI score changes among FHP patients. However, no significant effect was found (SMD = −0.812; 95% CI: −2.139 to 0.515; I^2^ = 84.1%, *p* = 0.0018, n = 91) (Figure 7). Two studies examined the impact of breathing exercises on NDI score alterations among patients with neck pain, and no substantial effect was found (SMD = −0.706; 95% CI: −1.893 to 0.480; I^2^ = 84.6%, *p* = 0.0109, n = 83) (Figure 7). Thus, the implementation of breathing exercises among patients with both FHP and CNP led to nonsignificant alterations in NDI scores compared to the controls (SMD = −0.747; 95% CI, −1.534 to 0.039; I^2^ = 79.8%, *p* = 0.0005, and n = 174).

#### 3.5.2. The Effects of Age Group on VAS, CVA, and NDI Among Patients with FHP and Neck Pain

**Figure 8:**
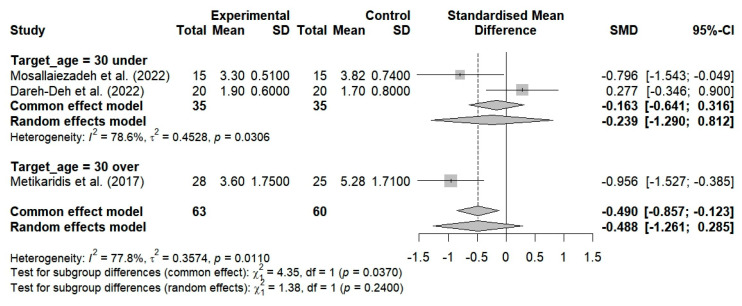
The effect of breathing exercise on VAS of subjects aged 30 years [20,21,22].

Two studies evaluated the effects of breathing exercises on the pain levels of subjects under the age of 30 and determined that there was no substantial improvement in pain (SMD = −0.239; 95% CI: −1.290 to 0.812; I^2^ = 78.6%, *p* = 0.0306, n = 70) (Figure 8). Furthermore, one study demonstrated that breathing exercises resulted in a substantial improvement in pain among subjects aged 30 years and older (SMD = −0.956; 95% CI: −1.527 to −0.385; n = 53) (Figure 8). The application of breathing exercises by age group thus showed nonsignificant changes in pain when compared to the controls (SMD = −0.488; 95% CI, −1.261 to 0.285; I^2^ = 77.8%, *p* = 0.0110, and n = 123) (Figure 8).

**Figure 9:**
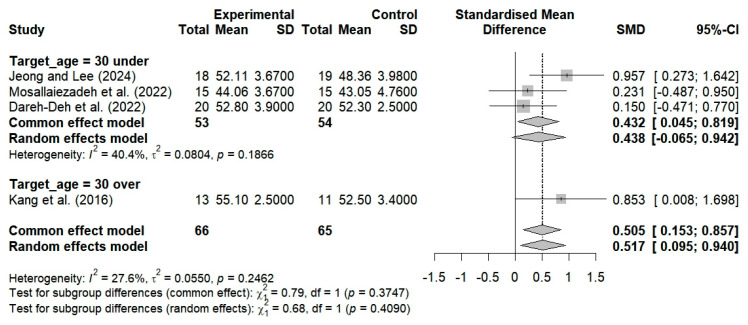
The effect of breathing exercise on CVA of subjects aged 30 years [19,21,22,23].

Three studies evaluated the efficacy of breathing exercises in altering neck alignment among subjects younger than 30 years of age. The findings revealed no significant changes in alignment (SMD = 0.438; 95% CI: −0.065 to 0.942; I^2^ = 40.4%, *p* = 0.1866, n = 107) (Figure 9). Furthermore, one study showed that neck alignment was substantially enhanced following breathing exercises among subjects aged 30 years and older (SMD = 0.853; 95% CI: 0.008 to 1.698; n = 24) (Figure 9). The implementation of breathing exercises tailored to different age groups thus resulted in substantial alterations in neck alignment when compared to the controls (SMD = 0.517; 95% CI: 0.095 to 0.940; I^2^ = 27.6%, *p* = 0.2462, n = 131) (Figure 9).

**Figure 10:**
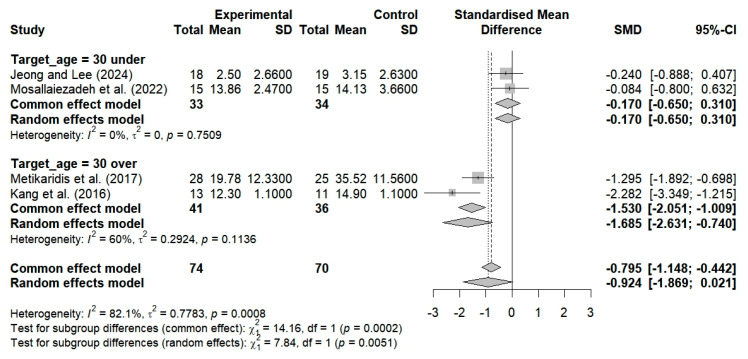
The effect of breathing exercise on NDI of subjects aged 30 years [19,20,22,23].

The NDI was used in two studies to evaluate neck dysfunction and the effects of breathing exercises among subjects under 30 years of age. These studies revealed no substantial alterations in the NDI scores (SMD = −0.170; 95% CI: −0.650 to 0.310; I^2^ = 0%, *p* = 0.7509, and n = 67) (Figure 10). In contrast, two studies revealed substantial enhancements in NDI scores following the administration of breathing exercises among subjects aged 30 years and older (SMD = −1.685; 95% CI: −2.631 to −0.740; I^2^ = 60%, *p* = 0.1136, n = 77) (Figure 10). The implementation of breathing exercises by age categories thus resulted in nonsignificant alterations in NDI scores when compared to the controls (SMD = −0.924; 95% CI: −1.869 to 0.021; I^2^ = 82.1%, *p* = 0.0008, n = 144) (Figure 10).

#### 3.5.3. The Impact of Varying Degrees of Neck Dysfunction on VAS, CVA, and NDI Among Patients Diagnosed with FHP and Neck Pain

**Figure 11:**
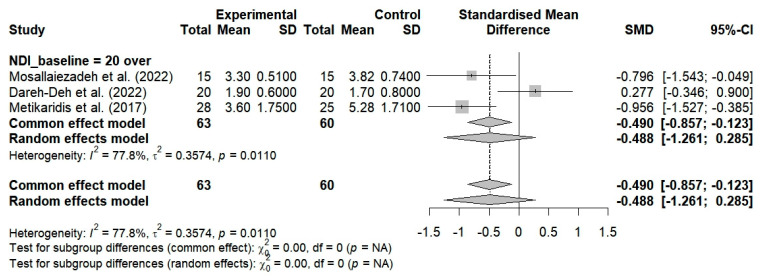
The effect of breathing exercise on VAS of subjects based on NDI 20 scores [20,21,22].

**Figure 12:**
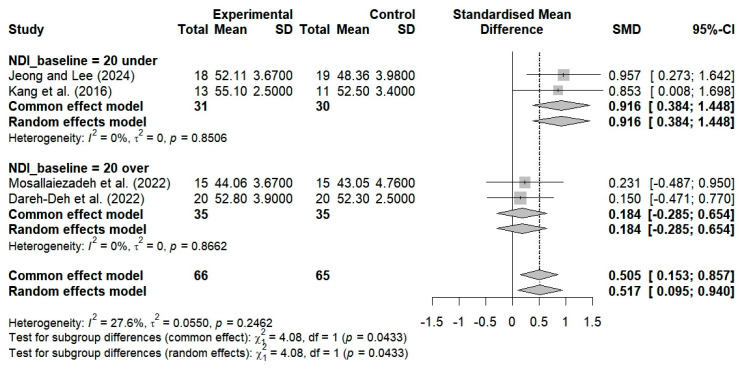
The effect of breathing exercise on CVA of subjects based on NDI 20 scores [19,21,22,23].

Three studies evaluated the pain levels of subjects with NDI scores ≥ 20 who performed breathing exercises. No significant improvements were observed (SMD = −0.488; 95% CI: −1.261 to 0.285; I^2^ = 77.8%, *p* = 0.0110, n = 123) (Figure 11). Two studies evaluated the cervical alignment of participants with NDI scores ≥ 20 who performed breathing exercises. No significant improvements were observed (SMD = 0.184; 95% CI: −0.285 to 0.654; I^2^ = 0%, *p* = 0.8662, n = 70) (Figure 12). Additionally, two studies that involved the use of breathing exercises for subjects with NDI < 20 showed significant improvements in neck alignment (SMD = 0.916; 95% CI: 0.384 to 1.448; I^2^ = 0%, *p* = 0.8506, n = 61) (Figure 12). The breathing exercises thus resulted in significant changes in neck alignment when applied to patients with NDI scores within the cutoff of 20 points, as compared to the controls (SMD = 0.517; 95% CI, 0.095 to 0.940; I^2^ = 27.6%, *p* = 0.2462, and n = 131) (Figure 12).

**Figure 13:**
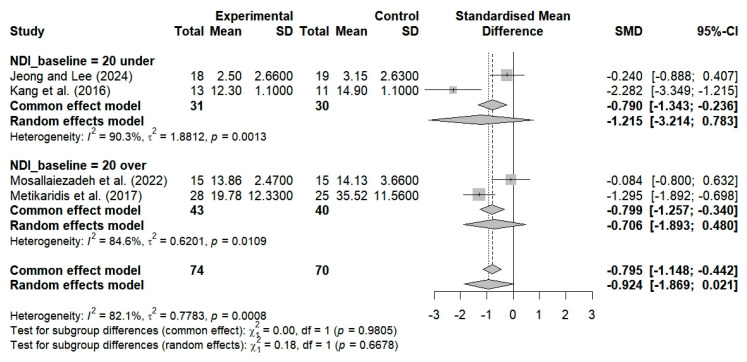
The effect of breathing exercise on NDI of subjects based on NDI 20 scores [19,20,22,23].

Two studies evaluated neck dysfunction by considering the effects of breathing exercises on participants with NDI scores ≥ 20 points. No significant improvement was observed (SMD = −0.706; 95% CI: −1.893 to 0.480; I^2^ = 84.6%, *p* = 0.0109, n = 83) (Figure 13). Additionally, two studies showed that breathing exercises resulted in nonsignificant changes in neck dysfunction among subjects with NDI scores below 20 (SMD = −1.215; 95% CI: −3.214 to 0.783; I^2^ = 90.3%, *p* = 0.0013, n = 61) (Figure 13). Analyzing the application of breathing exercises based on neck dysfunction severity using an NDI cutoff score of 20 showed no significant changes in NDI scores, as compared to the controls (SMD = −0.924; 95% CI: −1.869 to 0.021; I^2^ = 82.1%, *p* = 0.0008, n = 144) (Figure 13).

### 3.6. Publication Bias

**Figure 14:**
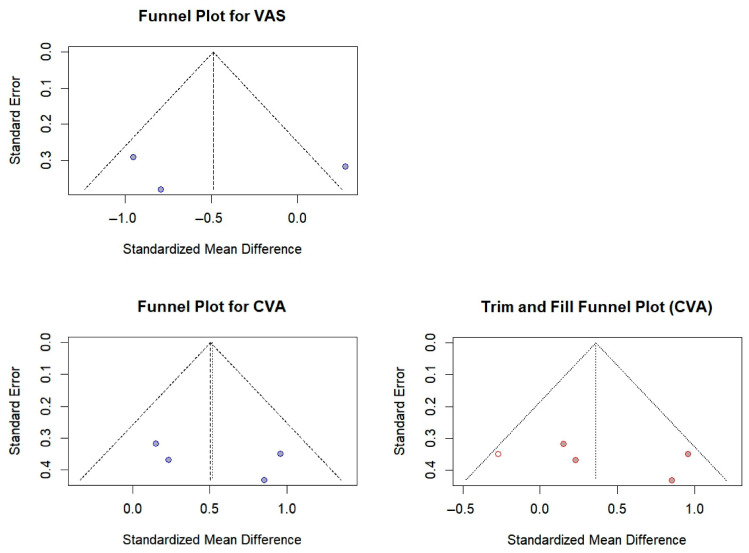

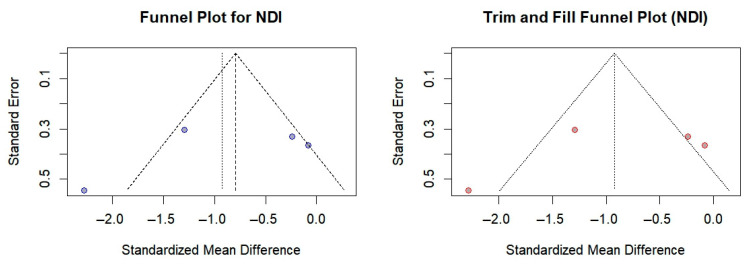
Funnel plots of the included studies.

Publication bias was examined visually with a single funnel plot figure (Figure 14) and statistically with the trim-and-fill procedure. For every outcome, fewer than ten studies were available (VAS = 3, CVA = 4, NDI = 4); therefore, Egger’s regression test was not performed because of its low statistical power under these conditions.

For VAS, Figure 14 appeared largely symmetrical, and trim-and-fill added no studies. The pooled effect size therefore remained SMD = −0.49 (95% CI −1.26 to −0.29; *p* = 0.0110; I^2^ = 77.8%), implying a low likelihood of publication bias for this outcome [20,21,22].

For CVA, the corresponding funnel plot in Figure 14 displayed some asymmetry. Trim-and-fill imputed one study, yielding an adjusted effect size of SMD = 0.3611 (95% CI −0.0872 to 0.8094; *p* = 0.1144). Although this correction reduced the magnitude of the original positive effect, it did not achieve statistical significance, suggesting that any publication bias present had little practical impact [19,21,22,23].

For NDI, Figure 14 shows slight visual asymmetry, yet trim-and-fill did not impute additional studies, indicating that publication bias was minimal. The pooled random-effects estimate remained SMD = −0.92 (95% CI −1.87 to 0.02; *p* = 0.0554; I^2^ = 82.1%), unchanged after adjustment [19,20,22,23].

Taken together, the visual inspections and trim-and-fill results provide little evidence that publication bias materially influenced the meta-analytic estimates for any of the three outcomes.

### 3.7. Grade Assessment

The certainty of the evidence for each outcome was assessed by two independent reviewers (SR and MB) using the Grading of Recommendations Assessment, Development, and Evaluation (GRADE) approach. The GRADE assessment considered five domains: risk of bias, consistency, directness, precision, and publication bias. The results are summarized in Appendix C. The certainty of the evidence for all outcomes ranged from very low to low. This likely reflects the methodological limitations of the included studies, significant statistical heterogeneity, and limited precision due to small sample sizes.

### 3.8. Sensitivity Analysis

#### 3.8.1. Leave-One-out Analysis

**Figure 15:**
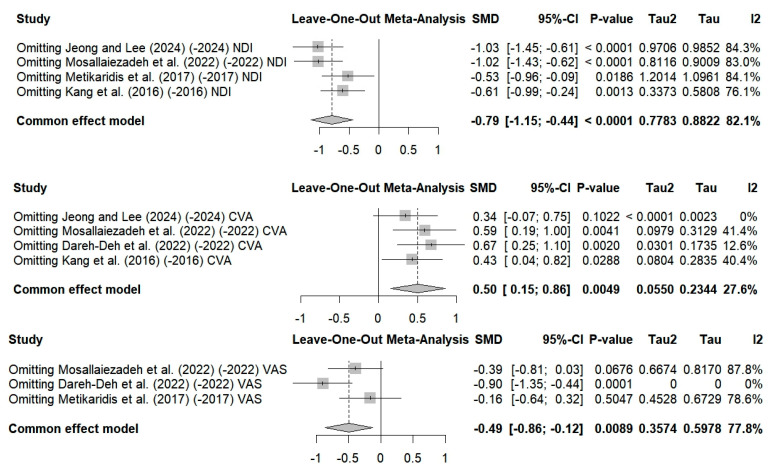
Leave-one-out analysis forest plots of the included studies [19,20,21,22,23].

A leave-one-out sensitivity analysis was performed to evaluate the impact of each study on the overall effect size for each outcome variable. All sensitivity analysis results are presented in Figure 15. When the NDI analysis results of Kang et al.’s study [19] were excluded, the effect size to SMD = −0.6121 (95% CI: −0.9863 to −0.2380, *p* = 0.0013) and heterogeneity was reduced (τ^2^ = 0.3373, I^2^ = 76.1%). Nevertheless, the direction of the overall effect remained consistent, confirming the stability of the results.

The results of the CVA analysis showed that removing any single study did not alter the direction of the overall intervention effect, which remained consistent at an SMD of 0.34–0.67. However, when Jeong et al.’s study [23] was excluded, the statistical significance decreased (SMD = 0.3423, 95% CI: −0.0682 to 0.7528, *p* = 0.1022) and heterogeneity decreased to 0% (τ^2^ < 0.0001, I^2^ = 0%). These results suggest that the study had a significant impact on the effect size and heterogeneity of the overall analysis. Further, excluding Dareh-Deh et al.’s study [21] caused the overall effect size increase to SMD = 0.6735 (95% CI: 0.2461 to 1.1010, *p* = 0.0020), and there was a tendency toward lower heterogeneity (I^2^ = 12.6%). This finding suggests that the study had a conservative impact on the overall analysis.

In the sensitivity analysis for VAS, the exclusion of Dareh-Deh et al.’s study [21] resulted in the most significant change. The effect size increased to SMD = −0.8970 (95% CI: −1.3510 to −0.4431, *p* = 0.0001), and heterogeneity was completely eliminated (I^2^ = 0%). This indicates that the study had unique characteristics that set it apart from other studies and that it served as the primary source of overall heterogeneity.

Conversely, the exclusion of Metikaridis et al.’s [20] study led to the loss of statistical significance (SMD = −0.1629, *p* = 0.5047) and a reduction in heterogeneity to a moderate level (I^2^ = 78.6%). Excluding Mosallaiezadeh et al.’s study [22] caused the effect size to diminish to SMD = −0.3927 (95% CI: −0.8138 to 0.0285) and become statistically insignificant (*p* = 0.0676).

#### 3.8.2. The Clinical Significance of the Sensitivity Analysis Merits Close Examination

The findings of the sensitivity analysis showed that the overall effect direction remained consistent, notwithstanding the heterogeneity in the study designs and the subject characteristics among the included studies. Notably, Dareh-Deh et al.’s study [21] demonstrated a positive effect at the individual level, and when incorporated into the meta-analysis, it served as an agent that augmented heterogeneity. This phenomenon is attributable to the distinctive three-group design and the study’s youthful demographic.

## 4. Discussion

This systematic review and meta-analysis comprehensively evaluated the clinical effectiveness of rehabilitation programs that included and did not include breathing interventions in patients with CNP and FHP. The results of this study showed that breathing interventions had a positive effect on improving cervical spine alignment (CVA) but did not significantly affect pain intensity (VAS score) or neck dysfunction (NDI score). Subgroup analysis also showed a tendency for statistically significant improvements in cervical spine alignment in FHP patients, as well as in pain intensity and neck dysfunction in patients aged 30 years and older; however, the overall certainty of this evidence was rated as ‘very low certainty’ in the GRADE assessment. Furthermore, significant improvements in cervical spine alignment were observed in patients with an NDI score of less than 20, suggesting that breathing interventions may influence postural and functional changes through broader mechanisms beyond simply improving breathing function.

### 4.1. Main Findings and Biomechanical and Neuromuscular Mechanisms

The most salient finding in this meta-analysis was that the breathing interventions resulted in significant improvements in the CVA of subjects with both CNP and FHP. The subgroup analysis revealed a statistically significant improvement in the FHP group, but no significant results were observed in the CNP group.

FHP is a postural abnormality characterized by reduced lordosis of the lower cervical spine and increased kyphosis of the upper thoracic spine. If persistent, this condition can lead to abnormal changes in thoracic shape and impairments in breathing function [44]. According to Kim et al. [45], the more severe the FHP, the greater the reduction in breathing function, and this is accompanied by an excessive use of abnormal respiratory accessory muscles. Specifically, as the head moves forward, the sternocleidomastoid muscle (SCM) remains in a state of continuous contraction [46], and respiratory accessory muscles that are typically activated at high inspiratory pressures (20 cmH_2_O or higher) are easily mobilized even at low inspiratory pressures [47]. Repeated abnormal breathing patterns accompanied by SCM overactivity cause tension and pain in the muscles around the neck, which solidifies FHP while reducing the activity of the diaphragm, which plays a leading role in normal breathing [48].

The interventions associated with significant improvements in CVA in this study primarily involved diaphragmatic breathing or feedback breathing. Many relevant prior studies have demonstrated that diaphragmatic breathing interventions, when applied for treatment, improve diaphragmatic function and alleviate the activity of neck muscles, including the SCM [22,23]. These previous findings suggest that the diaphragm-centered abdominal breathing intervention applied in this study contributed to normalizing abnormal breathing patterns and effectively reduced compensatory activity of neck muscles by shifting the primary role of breathing to the diaphragm [49]. Furthermore, Dareh-Deh et al. [21] and Kang et al. [19] have demonstrated that CVA improvements can be achieved through tool-based breathing exercises involving expert feedback. The findings of the present study suggest that feedback-based breathing exercises effectively suppress compensatory activity by providing appropriate load during inhalation and exhalation, ultimately leading to a reduction in SCM hyperactivity.

The specific mechanism by which normalizing this breathing pattern leads to CVA improvements can be explained as follows. First, the focus is on core stability and spinal alignment mechanisms. According to Hodges et al. [50], the diaphragm undergoes contraction during periods of rapid postural adjustment and thus plays a pivotal role in the process of postural stabilization. Masroor et al. [51] reported that diaphragmatic breathing movements increase intra-abdominal pressure and improve lumbar stability. This enhancement in core stability mitigates the load on the cervical spine, preventing the head from protruding further forward—a phenomenon particularly salient in FHP patients, whose upper thoracic cage is already expanded.

Second, the interaction between thoracic mechanics and spinal alignment must be considered. Vostatek et al.’s magnetic resonance imaging study [52] revealed that proper diaphragmatic function is essential for posture control. An et al. [13] reported that diaphragmatic breathing combined with cervical joint mobilization immediately improved the CVAs of stroke patients and explained that increased rib movement and improved posture alignment can be achieved through cervical spine realignment.

Third, the focus shifts to neurological mechanisms and fascial connectivity. According to Yekzaman et al.’s narrative literature review [53], there exists a possible association between cervical spine nerve compression and diaphragmatic dysfunction. An et al. [13] explained that joint mobilization applied to the cervical spine promotes the diaphragmatic nerve through spinal nerve expansion and influences the activation of the descending pathway, thereby increasing diaphragmatic contraction. Haghighat et al. [54] reported that diaphragmatic myofascial release provides additional value to forward head posture and thoracic expansion.

Finally, postural compensation mechanisms and whole-body alignment have been identified as critical factors in this process. Peng et al. [55] conducted a descriptive literature review and presented the pathophysiology, clinical evaluation, and management of cervical proprioceptive impairment in cases of neck pain. The authors suggested that breathing exercises may contribute to proprioceptive improvement. These multidimensional mechanisms are thought to work in concert to lead to significant improvement in CVA with breathing intervention in FHP patients.

The observation that the breathing interventions did not substantially improve CVA in the CNP group may be attributable to the intrinsic disparities between FHP and CNP. Fernández-de-las-Peñas et al. [56] reported a high prevalence of myofascial trigger points in neck and shoulder-related disorders, and Dimitriadis et al. [57] and Lasselin et al. [58] reported high prevalence rates of anxiety and depression among patients with chronic neck pain and explained that various psychosocial factors can complexly influence neck pain and functional impairment. Further, Shahidi et al.’s prospective cohort study [59] showed that depressive mood is the strongest predictor of chronic neck pain onset. This finding indicates that CNP is associated with intricate pathophysiological mechanisms that extend beyond rudimentary structural concerns. Consequently, we postulate that CNP is a multifaceted problem that cannot be effectively addressed solely through breathing interventions.

### 4.2. Limitations in Improving Pain and Functional Impairment and the Need for a Multifaceted Approach

The findings of this meta-analysis showed that the breathing interventions considered led to substantial enhancements in CVA but no statistically significant improvements in VAS and NDI. The GRADE assessment determined the certainty of this evidence to be very low, which can be partially explained by the high heterogeneity of the study results (I^2^ = 77.8% for VAS, I^2^ = 82.1% for NDI). Among the studies included in the meta-analysis on pain, Dareh-Deh et al.’s study [21] demonstrated a relatively low effect size, and this was identified as the primary cause of high heterogeneity. Moreover, in the sensitivity analysis of VAS, excluding the Dareh-Deh study resulted in an increased effect size of −0.8970, which was a statistically significant result (*p* = 0.0001), and heterogeneity was completely eliminated. These results suggest that the study’s design may have compromised its ability to demonstrate consistent pain reduction effects.

All studies that incorporated meta-analyses of NDI outcomes exhibited consistent negative effects (SMD range: −1.03 to −0.53), and effect size and heterogeneity declined, with the exception of Kang’s study [19]. Therefore, this particular study may have been a contributing factor to the variability in NDI score changes. This observation is analogous to the findings reported in Dareh-Deh’s study on VAS [21].

A further point that merits attention is the necessity of in-depth discussion when considering the complex characteristics of the measurement tools and the resulting specificity of breathing movements. The pain and functional impairments identified in this study were measured using the VAS and NDI. Given that the participants did not have pain caused by specific underlying conditions, it is possible that there were individual differences in the baseline pain levels. Furthermore, limitations resulting from individual differences in pain perception may have been present due to the subjective nature of the measurement tools used [60]. The evaluation items for the NDI encompass a range of factors, including pain, function, and psychosocial factors, which are intricately incorporated into 10 distinct items [61]. While the impacts of breathing exercises on physical functions related to postural stability have been well-documented, information on their direct association with cognitive functions or task performance in specialized environments, such as driving, is limited [62,63]. The effects of the breathing exercises considered in the present study may have offset the overall NDI score due to differential responses to NDI items. Subsequent studies should aim to examine changes in physical and cognitive function separately.

The substantial heterogeneity observed in the VAS (I^2^ = 77.8%) and NDI (I^2^ = 82.1%) results may have been caused by various clinical factors. A notable distinction emerged in the intervention protocols employed across the studies, underscoring the need for standardization in research methodologies to ensure comparability and reliability of findings. A series of observations were made with regard to the participants’ respiratory techniques, which included diaphragmatic breathing, resistance breathing, and feedback breathing. Additionally, the duration of the intervention was noted, ranging from two to eight weeks. Concomitant therapeutic interventions, such as McKenzie exercises, shoulder stabilization exercises, and standard physical therapy, were also documented. The characteristics of the study populations also varied by gender or age [22,23]. The observed statistical heterogeneity can be attributed, at least in part, to the documented differences among the studies that were included in the analysis. This observation underscores the necessity for individualized treatment approaches in clinical practice.

### 4.3. Differential Effects and Clinical Implications of the Subgroup Analyses

The results of the subgroup analyses showed that the effects of breathing interventions may vary depending on the baseline characteristics of the patients. Significant guidelines for the individualization of rehabilitation interventions could be determined from the findings.

#### 4.3.1. Effect by Age

In this meta-analysis, the effects of the breathing exercises were analyzed by age group. The results indicated improvements in CVA (SMD = 0.85), NDI (SMD = 0.17), and VAS (SMD = 0.96) among adults aged 30 years and older. Despite the inherent limitations of the study, we included the analysis of only a single study for CVA and VAS and the NDI analysis (SMD = −1.685) involving two groups. While statistically significant improvements were observed in pain, alignment, and function contingent on age, given the ‘very low certainty’ of this GRADE evidence, its clinical significance should be cautiously considered as a potential hypothesis. In the general population, factors such as postural imbalance, stress, and reduced body awareness tend to accumulate after the age of 30. These factors can lead to a progressive decrease in breathing system compliance due to rib calcification, thoracic kyphosis progression, and reduced diaphragm strength, resulting in a shallow breathing pattern centered in the upper thoracic region and, in turn, reduced breathing efficiency [36,37,38,39,40]. The analyzed studies that involved participants aged 30 or older were those by Kang et al. [19] and Metikaridis et al. [20], where the experimental groups were in their 50s and mid-30s on average, respectively. As shown in previous studies, it seems that age-related changes may have progressed more in older subjects [36,37,38,39,40]. The severity of these changes is positively correlated with the effectiveness of diaphragm-centered deep breathing as an intervention for various complex issues related to the neck. This finding has a significant clinical implication: Breathing interventions can serve as strategic approaches that go beyond merely improving breathing function for middle-aged individuals with complex issues (e.g., posture, pain, and function). These findings suggest that breathing interventions may be most beneficial for adults aged 30 and over, possibly as preventive strategies. However, given the very low certainty of the evidence, its clinical application should be considered experimental and require explicit informed consent regarding the limitations of the evidence.

#### 4.3.2. Effects According to the Level of Basic Functional Impairment (Initial NDI Value)

The CVA improvement effect was robustly observed in the mild disability group with an initial NDI score of <20 points, with an SMD of 0.92 (GRADE: low). No significant effect was observed in the severe disability group. These results suggest that the impact of breathing exercises may vary depending on the severity of functional impairment.

The substantial CVA enhancement seen in the mild disability group substantiates the prevailing notion that breathing exercises exert disparate effects on physical and cognitive functions. Hagins et al. [62] reported that breathing control directly affects intra-abdominal pressure during lifting tasks, and they suggested that breathing exercises primarily affect structural and postural aspects. The lack of a significant improvement in the severe disability patient group following breathing exercise was likely due to the correlation between high NDI scores and the presence of complex issues [30,64]. The NDI encompasses a wide range of domains, including pain intensity, personal management, work performance, and cognitive activities. This comprehensive approach enables the identification of patients who are likely to experience complex functional impairments beyond simple postural abnormalities. The abovementioned findings indicate the need for a multimodal approach to neck pain management and corroborate the significance of the clinical practice guidelines established by Bussières et al. [65], which advocate for a multimodal approach that integrates a range of interventions for the management of acute or chronic neck pain.

The present study had certain limitations, most notably the inability to analyze the pain period in detail. However, the findings of our analysis confirm that the greater the degree of functional disability, the more difficult it is to achieve substantial improvement with merely a simple breathing intervention. Conversely, for patients with mild functional impairment, simple breathing interventions may be potentially effective for changes in alignment. The findings of this study underscore the significance of preventive measures, such as breathing exercises, in cases of mild functional disability. Subsequent studies should assess the efficacy of preventive interventions and various intervention approaches according to the severity of functional disability. The preferential response observed in mild disability patients suggests that breathing interventions may be the most cost-effective form of early intervention. Simple techniques require minimal resources; however, standardized protocols are necessary prior to their routine implementation.

#### 4.3.3. Comparative Analysis of the Breathing Intervention Methods

The primary features and implications of the breathing intervention methods presented in this study are as follows: Diaphragmatic breathing has been a subject of considerable research. The majority of studies have focused on diaphragm-centered breathing, also known as abdominal breathing. This approach is pivotal in addressing the restricted chest movement and diminished diaphragm efficiency frequently observed in patients with FHP, as it helps reduce the overuse of accessory breathing muscles. It has also been shown to alleviate tension and discomfort in the neck muscles. For instance, in Jeong and Lee’s study [23], diaphragmatic breathing retraining effectively reduced excessive neck muscle activity and restored diaphragmatic function.

The utilization of various techniques and tools is imperative for the effective management of CNP. Kang et al. [19] employed a feedback device (SPIROTIGER^®^), Dareh Deh et al. [21] utilized balloons, and Mosallaiezadeh et al. [22] provided visual/tactile feedback to facilitate the recognition of breathing patterns and the acquisition of proper techniques. This highlights a potential avenue for enhancing the efficacy of breathing interventions. Feedback enables patients to develop an objective understanding of their own breathing patterns, which enhances their capacity to precisely modulate diaphragm movement and make abdominal pressure adjustments. This facilitates the acquisition of precise and efficient breathing patterns.

The following is a list of rehabilitation exercises that can be combined with diaphragmatic breathing: In the majority of studies, breathing interventions were not utilized in isolation but in conjunction with other physical therapy and exercise programs, including shoulder stabilization exercises [23], general physical therapy [22], McKenzie exercises [19], and stretching and strength training [21]. This indicates that breathing interventions are integral components of comprehensive rehabilitation programs that aim to achieve synergistic effects. Indeed, Mosallaiezadeh et al. [22] reported sustained effects in reducing pain intensity and improving cervical dysfunction when diaphragmatic exercises were combined with physical therapy.

It is imperative to deliberate upon the implications of stress and psychosocial factors. In Metikaridis et al.’s study [20], diaphragmatic breathing was incorporated as a component of a stress management program that addressed psychosocial factors. The findings showed that the stress management program significantly reduced the pain intensity and functional impairment of patients with chronic neck pain. This emphasizes the importance of addressing psychological factors and indicates that, given the multifaceted nature of chronic pain, a psychological intervention may be necessary in conjunction with physical interventions.

Variability in intervention duration and frequency: The duration of the studies ranged from two to eight weeks, and the frequency of sessions varied from one to five sessions per week. This underscores the need for additional research to ascertain the optimal dosage and duration of breathing interventions.

The application of remote rehabilitation: In a noteworthy development, Jeong and Lee [23] pioneered the application of breathing intervention through remote rehabilitation during the pandemic. This development has been shown to enhance accessibility, reduce temporal and spatial constraints, and foster heightened patient motivation to engage in treatment regimens, thereby contributing to enhanced long-term treatment compliance. Furthermore, remote rehabilitation has been demonstrated to offer economic benefits and to be conducive to future technological development. It also has the potential to expand rehabilitation opportunities to patients beyond specific regions worldwide [66].

The wide array of breathing intervention approaches identified in the included studies may have contributed to the substantial heterogeneity in outcomes for pain (I^2^ = 77.8%) and functional impairment (I^2^ = 82.1%). The observed variations in therapeutic responses may be attributed to mechanistic differences across interventions, contingent upon the underlying objectives. The utilization of devices such as feedback devices or balloons, which aim to modify breathing patterns through real-time biofeedback [19,21], may result in more consistent neuromuscular adaptations compared to basic diaphragmatic breathing [20,23]. Furthermore, studies have shown that resistive breathing exercises, which are designed to strengthen respiratory muscles, may elicit different physiological adaptations than balloon breathing exercises involving volume limitations. Additionally, the integration of simple breathing exercises with other interventions exhibited a high degree of variability. Metikaridis et al. [20] incorporated diaphragmatic breathing into a stress management program, addressing both biomechanical dysfunction and psychosocial factors. The heterogeneity of these interventions, as evidenced by the elevated heterogeneity scores in the study’s outcomes, mirrors the clinical reality that respiratory interventions may necessitate individualization based on patient characteristics, available resources, and treatment objectives. The variation in efficacy patterns across different intervention types underscores the necessity of implementing customized breathing techniques, tailored to each patient’s symptoms, as opposed to a uniform approach.

#### 4.3.4. Comparison with Other Meta-Analyses on Breathing Interventions

A meta-analysis on breathing exercises and neck pain published in 2025 reported that the exercises facilitated significant pain reduction (SMD = −10.16) [14], but, like in the present study, the certainty of the evidence was low. Furthermore, a meta-analysis conducted in 2022 on breathing dysfunction among CNP patients showed a substantial decline in respiratory muscle strength and lung function (maximum inspiratory pressure: −11.67; maximum expiratory pressure: −11.80) [8], which corroborates the present study’s discourse on FHP curtailing thoracic movement and diminishing diaphragmatic efficiency, ultimately culminating in impaired breathing function. In Cefalì et al.’s recent meta-analysis [14], the evidence level was rated low due to heterogeneity and bias regarding the pain and functional improvement effects of breathing training. This finding is consistent with the limitation of the high heterogeneity of VAS and NDI results in the present study. A systematic review by Tatsios et al. [67] that included 16 randomized controlled trials showed that spinal and diaphragmatic manual therapy, stabilization exercises, and breathing exercises improved musculoskeletal and breathing outcomes. However, high heterogeneity among the studies was noted, and the certainty of the evidence was rated low or very low.

#### 4.3.5. Incremental Value and Distinctiveness Compared to Previous Reviews

Firstly, the systematic approach adopted in this study is congruent with the theoretical underpinnings of CNP and FHP. The two conditions share complex pathophysiological mechanisms that influence each other. The integrated effects of breathing interventions were evaluated by addressing CNP and FHP together.

Secondly, in order to quantify the independent contribution of respiratory interventions, a clear definition of the presence and absence of respiratory interventions was established, and comparative analyses were conducted based on this definition. This finding lends further credence to the notion that the incorporation of respiratory components into rehabilitation programs can yield significant benefits.

Thirdly, by systematically assessing the heterogeneity of intervention effects using predefined subgroup analyses, such as the GRADE approach, we were able to address more nuanced details beyond general effectiveness assessments. The present study offers a foundation for the selection of customized treatment regimens, as opposed to a uniform approach, by means of the identification of differential effects based on patient characteristics such as age and level of functional impairment. These results signify a substantial methodological and clinical advancement in the application of respiratory interventions for cervical-related ailments.

The findings of this study suggest the potential significance of breathing re-education in physical therapy practice. In the context of FHP patients, breathing interventions should be considered potentially important therapeutic components that can help reduce SCM hyperactivity and enrich diaphragmatic function. A multidisciplinary approach is crucial for considering complex aspects such as central sensitization, psychological factors, and cervical proprioceptive abnormalities, in addition to biomechanical correction for the complete resolution of chronic pain and functional impairment. Breathing interventions should not be utilized as standalone treatment modalities but should instead be incorporated as a fundamental component into a comprehensive rehabilitation program. This approach can prevent compensatory mechanisms and facilitate the restoration of appropriate muscle contraction and relaxation capabilities.

However, it should be acknowledged that the expansion from standalone breathing exercises to comprehensive rehabilitation programs incorporating breathing interventions introduced methodological complexity that limits the precision of our conclusions regarding the independent effects of breathing components. While this approach enhanced the clinical relevance of our findings by reflecting real-world practice patterns, it compromised the ability to make definitive statements about the isolated therapeutic value of breathing interventions.

#### 4.3.6. Study Limitations and Future Research Suggestions

This meta-analysis has several limitations. Firstly, the number of included studies was limited, thereby reducing the overall sample size. This may increase the risk of Type II error, i.e., failure to detect true effects when they exist, for some outcomes that did not reach statistical significance (e.g., overall effects of VAS and NDI). Second, a high degree of heterogeneity was observed across studies, likely due to differences in methodology and participant characteristics across RCTs. For example, the included studies varied in patient age, gender, and presence of FHP, as well as the type of breathing training (e.g., diaphragmatic breathing vs. respiratory muscle strengthening), intensity, and duration. One study [23] included only young men, while another [22] included only women, creating differences in the study populations. The presence of heterogeneity factors may have resulted in increased variability in the overall results and a concomitant weakening of statistical significance. Furthermore, a substantial degree of heterogeneity was observed in the results for pain (VAS) and functional disability (NDI). A number of the included studies demonstrated deficiencies in their methodological quality. For instance, the studies by Metikaridis et al. [20] and Jeong and Lee [23] exhibited a high risk of bias (high risk) in the RoB 2.0 assessment, which may have resulted in an overestimation of the results. In raising concerns about potential bias, Kang et al. [19] emphasized the necessity of meticulous verification. In Cefalì et al.’s recent meta-analysis [14], breathing exercises were reported to improve pain and function; however, due to heterogeneity and bias, the evidence level was judged to be low. This study’s GRADE assessment determined that the level of evidence for improvements in pain, disability, and posture was very low. This is likely due to the limited number of studies included in the analysis for each outcome measure (VAS: 3; NDI: 4; CVA: 4), which introduced imprecision and resulted in a lower GRADE rating. The “very low certainty” GRADE rating indicates very low confidence in the effect estimate and may influence clinical decision-making, requiring further discussion. It should be noted that the clinical application of breathing interventions based on these results is experimental. Additional large-scale, high-quality randomized controlled trials (RCTs) and studies with sufficient follow-up are needed to upgrade the certainty of the evidence.

Third, the repeated utilization of data from the same study in the subgroup analysis by diagnosis was a significant limitation. A number of RCTs involved both CNP and FHP patients within a single study, and these were included in the present review for separate comparisons. This methodological flaw may have led to artificially inflated precision in confidence intervals and potentially biased effect estimates. A random-effects model was employed in this study to address the correlation issues arising from such duplication in the meta-analysis. However, it is plausible that residual correlations between effect values derived from the same study were not fully eradicated. When interpreting the effect sizes and confidence intervals resulting from the subgroup analysis, which demonstrated high heterogeneity (pain VAS: I^2^ = 77.8%; functional disability NDI: I^2^ = 82.1%), it is imperative to consider the potential for bias, as the precision of the results may have been overestimated. Posture alignment (CVA) exhibited comparatively low heterogeneity (I^2^ = 27.6%). This may be attributable to the properties of the measurement instruments or the uniformity of the intervention effects. The potential for unit-of-analysis errors to exert a statistically negligible influence cannot be discounted.

Fourth, the expansion of the research scope from standalone breathing exercises to ‘rehabilitation programs incorporating breathing interventions’ represents a significant methodological limitation. This pragmatic adjustment was necessitated by the scarcity of studies focusing solely on breathing interventions, but it introduced substantial challenges in determining the pure effect of breathing components. The complex interactions between breathing interventions and concurrent treatments (such as McKenzie exercises, shoulder stabilization, and standard physiotherapy) made it difficult to isolate the independent contribution of respiratory components. The observed high heterogeneity in pain (I^2^ = 77.8%) and functional disability outcomes (I^2^ = 82.1%) may be partially attributed to this methodological complexity, as different combinations of interventions across studies precluded definitive conclusions about the specific therapeutic value of breathing interventions. Future studies should prioritize investigating standalone breathing interventions with appropriate control groups to establish more definitive evidence regarding their independent therapeutic efficacy.

Future studies should consider the following directions. First, there is a need for RCTs with larger sample sizes and rigorous study designs that include efforts to minimize bias risks, such as double blinding. Second, long-term follow-up studies are imperative for evaluating the long-term sustainability of pain, functional impairment, and posture improvement effects. Third, studies need to directly explore the effects of breathing interventions on central pain processing and psychological factors. Such interventions could include brain imaging techniques and psychosocial indicators. Furthermore, qualitative research methodologies (e.g., focus groups and in-depth interviews) should be employed to explore patients’ perspectives on the use of breathing exercises for neck pain (e.g., treatment expectations, beliefs, and experiences) to obtain information on intervention feasibility and acceptability in clinical settings [21,68]. Fourth, studies should employ objective, detailed measurement tools for breathing function (e.g., lung capacity and respiratory muscle strength) and cervical proprioception to elucidate the mechanistic effects of breathing interventions. Fifthly, it is imperative to extend the ambit of research in order to encompass a more heterogeneous array of groups. It is imperative to ascertain the efficacy of breathing intervention programs in groups that have not been previously identified, such as adolescents, the elderly, and gender-based differences, to enhance the external validity (generalizability) of research findings. Sixth, the limited number of included studies precluded the use of meta-regression analysis to explore potential moderating factors such as intervention duration, breathing training types, or dosage parameters. Meta-regression analysis requires a minimum of 10 studies per covariate to ensure adequate statistical power, but our meta-analysis included only 3–4 studies per outcome. Future meta-analyses with larger numbers of included studies should employ meta-regression techniques to systematically investigate how intervention characteristics (duration, frequency, type of breathing technique, use of feedback devices, concurrent interventions) moderate treatment effects. Such analyses would provide more precise guidance for optimizing breathing intervention protocols and could help explain the substantial heterogeneity observed in our results. Finally, future meta-analyses should use more sophisticated statistical methods, such as robust variance estimation or multilevel meta-analysis, to appropriately address the statistical dependence of effect sizes from the same study.

## 5. Conclusions

This meta-analysis demonstrated that a rehabilitation program incorporating breathing interventions could significantly improve cervical spine alignment (CVA) and posture in patients with CNP and FHP. Specifically, breathing interventions were shown to induce diaphragmatic use, thereby restoring diaphragmatic function, reducing compensatory activity of accessory respiratory muscles, and promoting sensorimotor integration relearning. While insignificant effects on pain and functional disability were observed, this may be due to the complex nature of chronic pain (i.e., central hypersensitivity, psychological factors, and sensorimotor mismatch). These results suggest that breathing interventions, when combined with multifaceted interventions such as psychological interventions, may be beneficial in reducing pain and improving function. Clinical benefits were particularly observed in patients with mild functional disability and in adults aged 30 years or older. These findings highlight the importance of tailoring breathing intervention strategies to individual patient characteristics.

These findings strongly suggest that breathing re-education is a useful tool in physical therapy for the correction of FHP, as it supports the spine from below and relieves pathological upper extremity tension. However, comprehensive treatment for CNP, achieving meaningful pain relief and functional improvement, requires multidisciplinary interventions targeting both the central and psychological aspects of pain. Future research should focus on overcoming the limitations of this study and elucidating the long-term effects and mechanisms of breathing interventions to provide optimal evidence-based treatment guidelines for patients with CNP and postural imbalance.

## Figures and Tables

**Table 1:** PICOS eligibility inclusion/exclusion criteria.

Category	Inclusion Criteria
Population	Adults aged 18 years or older with chronic neck pain and/or FHP.
Intervention	Breathing interventions (e.g., diaphragmatic breathing, respiratory muscle training, feedback breathing, etc.), with a minimum duration of 2 weeks.
Comparator	Rehabilitation without breathing exercises (standard physical therapy, exercise therapy, posture correction programs, no treatment, or minimal intervention control groups).
Outcomes	At least one: neck pain intensity (VAS/NRS), neck disability (NDI), forward head posture (CVA or equivalent).
Study Design	Randomized controlled trials (RCTs), including pilot RCTs.
Other	English language publications. Date of publication: no restrictions.
**Reason**	**Exclusion Criteria**
1	Studies including participants under 18 or without chronic neck pain/FHP.
2	Specific pathologies (e.g., trauma, cancer, disk herniation, inflammatory diseases).
3	Interventions not related to exercise/rehabilitation (e.g., purely pharmacologic or surgical); breathing < 2 weeks or not central to the intervention.
4	Comparators including breathing exercises in both groups.
5	Non-RCTs, observational studies, case reports, protocols, reviews, book chapters, conference abstracts, unpublished theses.
6	Studies lacking VAS, NDI, and CVA data or having insufficient outcome data.
7	Duplicate data and poor methodology (e.g., inadequate randomization/allocation concealment).

Abbreviations: FHP, forward head posture; VAS, visual analog scale; NRS, numerical rating scale; NDI, neck disability index; CVA, cervical vertebrae angle; RCT, randomized controlled trial.

## Data Availability

The datasets used and analyzed during the current study are available from the corresponding author on reasonable request.

## Abbreviations

The following abbreviations are used in this manuscript:

CNP	Chronic neck pain
FHP	Forward head posture
VAS	Visual analog scale
NRS	Numeric rating scale
CVA	Craniovertebral angle
RCTs	Randomized controlled trials
NDI	Neck disability index
SMDs	Standardized mean differences
CIs	Confidence intervals
SCM	Sternocleidomastoid muscle

## Appendix A

Search Strategy

A comprehensive literature search was conducted across four electronic databases: PubMed, CINAHL, Embase, MEDLINE, Cochrane Library, Web of science and Scopus.

PubMed:

(“neck pain”[MeSH Terms] OR “chronic neck pain” OR “cervical pain” OR “cervicalgia” OR “forward head posture” OR “FHP” OR “head posture”)

AND

(“breathing exercises”[MeSH Terms] OR “diaphragmatic breathing” OR “deep breathing” OR “respiratory muscle training”)

AND

(“exercise therapy”[MeSH Terms]

OR “physical therapy modalities”[MeSH Terms]

OR “physiotherapy”

OR “manual therapy”

OR “rehabilitation”[MeSH Terms]

OR “therapeutic exercise”

OR “physical exercise”

OR “postural correction”

OR “

CINAHL:

((“neck pain” OR “chronic neck pain” OR “cervicalgia” OR “cervical pain” OR “forward head posture” OR “FHP” OR “head posture”)) AND ((“breathing exercises” OR “diaphragmatic breathing” OR “deep breathing” OR “respiratory muscle training” OR “breathing techniques” OR “respiratory training”)) AND ((“exercise therapy” OR “physical therapy” OR “rehabilitation” OR “manual therapy” OR “conventional therapy”)) NOT ((“systematic review” OR “meta-analysis” OR review))

Embase:

(‘neck pain’/exp OR ‘neck pain’ OR ‘chronic neck pain’/exp OR ‘chronic neck pain’ OR ‘cervical pain’/exp OR ‘cervical pain’ OR ‘cervicalgia’/exp OR ‘cervicalgia’ OR ‘forward head posture’/exp OR ‘forward head posture’ OR ‘fhp’ OR ‘head posture’/exp OR ‘head posture’) AND (‘breathing exercise’/exp OR ‘breathing exercise’ OR ‘diaphragmatic breathing’/exp OR ‘diaphragmatic breathing’ OR ‘deep breathing’/exp OR ‘deep breathing’ OR ‘respiratory muscle training’/exp OR ‘respiratory muscle training’ OR ‘breathing techniques’ OR ‘respiratory training’/exp OR ‘respiratory training’) AND (‘exercise therapy’/exp OR ‘exercise therapy’ OR ‘physical therapy’/exp OR ‘physical therapy’ OR ‘rehabilitation’/exp OR ‘rehabilitation’ OR ‘manual therapy’/exp OR ‘manual therapy’ OR ‘conventional therapy’/exp OR ‘conventional therapy’) NOT (‘systematic review’/exp OR ‘systematic review’ OR ‘meta analysis’/exp OR ‘meta analysis’ OR review:it)

MEDLINE:

1

neck pain/or chronic neck pain.mp. or cervicalgia.mp. or forward head posture.mp. or FHP.mp.

2

breathing exercises/or diaphragmatic breathing.mp. or respiratory muscle training.mp. or deep breathing.mp. or breathing retraining.mp.

3

exercise therapy/or physical therapy modalities/or physiotherapy.mp. or rehabilitation.mp.

4

1 and 2 and 3

Cochrane Library:

(“Forward Head Posture” OR “Craniovertebral Angle” OR “neck pain”) AND ([mh “Breathing Exercises”] OR “Breathing Exercise*” OR “Respiratory Training”) AND [mh “Randomized Controlled Trials as Topic”]

Web of science:

TS = (“neck pain” OR “forward head posture” OR “craniovertebral angle”)

AND TS = (“breathing exercise*” OR “respiratory training”)

AND TS = (“randomized controlled trial” OR “RCT”)

Scopus:

TITLE-ABS-KEY(“neck pain” OR “chronic neck pain” OR “cervical pain” OR “cervicalgia” OR “forward head posture” OR “fhp” OR “head posture”)

AND

(“breathing exercise” OR “diaphragmatic breathing” OR “deep breathing” OR “respiratory muscle training” OR “breathing techniques” OR “respiratory training”)

AND

(“exercise therapy” OR “physical therapy” OR “rehabilitation” OR “manual therapy” OR “conventional therapy”)

AND NOT

(“systematic review” OR “meta analysis” OR “review”)

## Appendix B

Key characteristics and citations of included studies.

This appendix contains tables of key characteristics of all studies included in the present systematic review.

Each study is presented in a separate table with a standardized format. If a cell is empty, the study authors did not sufficiently describe the characteristic.

**Table A1:** Characteristics of the included studies.

Study	Study Design	Number of Treated Necks	Post-Treatment Follow-Up	Outcome Measures
Kang et al. [19] 2016	RCT	24	2 weeks	CVA, NDI
Metikaridis et al. [20] 2017	RCT	53	8 weeks	VAS, NDI
Dareh-Deh et al. [21] 2022	RCT	60	8 weeks	VAS, CVA
Mosallaiezadeh et al. [22] 2022	RCT	30	2 weeks	VAS, CVA, NDI
Jeong and Lee. [23] 2024	RCT	37	4 weeks	CVA, NDI

Abbreviations: RCT, randomized controlled trial; CVA, cervical vertebrae angle; VAS, visual analog scale; NDI, neck disability index.

## Appendix C

**Table A2:** GRADE Evidence Summary.

Certainty Assessment							№ of Patients		Effect		Certainty	Importance
№ of Studies	Study Design	Risk of Bias	Inconsistency	Indirectness	Imprecision	Other Considerations	Brathing Exercise	Without Breathing Exercise	Relative (95% CI)	Absolute (95% CI)		
**All breathing exercise on Pain (VAS)**												
3	randomized trials	serious	very serious	not serious	very serious	none	63	60	-	SMD **0.488 SD lower** (1.261 lower to 0.285 higher)	⨁◯◯◯ Very low	CRITICAL
**All breathing exercise on CVA**												
4	randomized trials	serious	serious	not serious	serious	none	66	65	-	SMD **0.517 SD higher** (0.095 higher to 0.94 higher)	⨁◯◯◯ Very low	CRITICAL
**All breathing exercise on NDI**												
4	randomized trials	serious	very serious	not serious	very serious	none	74	70	-	SMD **0.924 SD lower** (1.869 lower to 0.021 higher)	⨁◯◯◯ Very low	CRITICAL
**FHP + breathing exercise on Pain (VAS)**												
2	randomized trials	not serious	very serious	not serious	very serious	none	35	35	-	SMD **0.239 SD lower** (1.29 lower to 0.812 higher)	⨁◯◯◯ Very low	CRITICAL
**CNP + breathing exercise on Pain (VAS)**												
3	randomized trials	serious	very serious	not serious	very serious	none	63	60	-	SMD **0.488 SD lower** (1.261 lower to 0.285 higher)	⨁◯◯◯ Very low	CRITICAL
**FHP + breathing exercise on CVA**												
4	randomized trials	serious	serious	not serious	serious	none	66	65	-	SMD **0.517 SD higher** (0.095 higher to 0.94 higher)	⨁◯◯◯ Very low	CRITICAL
**CNP + breathing exercise on CVA**												
4	randomized trials	not serious	not serious	not serious	very serious	none	35	6535	-	SMD **0.184 SD higher** (0.285 lower to 0.654 higher)	⨁⨁◯◯ Low	CRITICAL
**FHP + breathing exercise on NDI**												
3	randomized trials	serious	very serious	not serious	very serious	none	46	45	-	SMD **0.526 SD lower** (2.139 lower to 0.515 higher)	⨁◯◯◯ Very low	CRITICAL
**CNP + breathing exercise on NDI**												
2	randomized trials	serious	very serious	not serious	very serious	none	43	40	-	SMD **0.706 SD lower** (1.893 lower to 0.48 higher)	⨁◯◯◯ Very low	CRITICAL
**30 age under + breathing exercise on Pain (VAS)**												
2	randomized trials	not serious	very serious	not serious	very serious	none	35	35	-	SMD **0.239 SD lower** (1.29 lower to 0.812 higher)	⨁◯◯◯ Very low	CRITICAL
**30 age over + breathing exercise on Pain (VAS)**												
1	randomized trials	serious	not serious	not serious	very serious	none	28	25	-	SMD **0.488 SD lower** (1.261 lower to 0.285 higher)	⨁◯◯◯ Very low	CRITICAL
**30 age under + breathing exercise on CVA**												
3	randomized trials	serious	very serious	not serious	not serious	none	53	54	-	SMD **0.438 SD higher** (0.065 lower to 0.942 higher)	⨁◯◯◯ Very low	CRITICAL
**30 age over + breathing exercise on CVA**												
1	randomized trials	serious	very serious	not serious	serious	none	13	11	-	SMD **0.853 SD higher** (0.008 higher to 1.698 higher)	⨁◯◯◯ Very low	CRITICAL
**30 age under + breathing exercise on NDI**												
1	randomized trials	serious	not serious	not serious	very serious	none	33	34	-	SMD **0.17 SD lower** (0.65 lower to 0.31 higher)	⨁◯◯◯ Very low	CRITICAL
**30 age over + breathing exercise on NDI**												
2	randomized trials	serious	very serious	not serious	very serious	none	41	36	-	SMD **1.685 SD lower** (2.631 lower to 0.74 lower)	⨁◯◯◯ Very low	CRITICAL
**NDI score 20 over + breathing exercise on Pain (VAS)**												
3	randomized trials	serious	very serious	not serious	very serious	none	63	60	-	SMD **0.488 SD lower** (1.261 lower to 0.285 higher)	⨁◯◯◯ Very low	CRITICAL
**NDI score 20 under + breathing exercise on CVA**												
2	randomized trials	serious	not serious	not serious	serious	none	31	30	-	SMD **0.916 SD higher** (0.384 higher to 1.448 higher)	⨁⨁◯◯ Low	CRITICAL
**NDI score 20 over + breathing exercise on CVA**												
2	randomized trials	serious	not serious	not serious	very serious	none	35	35	-	SMD **0.184 SD higher** (0.285 lower to 0.654 higher)	⨁◯◯◯ Very low	CRITICAL
**NDI score 20 under + breathing exercise on NDI**												
2	randomized trials	serious	very serious	not serious	very serious	none	31	30	-	SMD **1.215 SD lower** (3.214 lower to 0.783 higher)	⨁◯◯◯ Very low	CRITICAL
**NDI score 20 over + breathing exercise on NDI**												
2	randomized trials	serious	very serious	not serious	very serious	none	43	40	-	SMD **0.706 SD lower** (1.893 lower to 0.48 higher)	⨁◯◯◯ Very low	CRITICAL

**CI**: confidence interval; **SMD**: standardized mean difference.
